# Bone Metastases in Neuroendocrine Neoplasms: From Pathogenesis to Clinical Management

**DOI:** 10.3390/cancers11091332

**Published:** 2019-09-08

**Authors:** Barbara Altieri, Carla Di Dato, Chiara Martini, Concetta Sciammarella, Antonella Di Sarno, Annamaria Colao, Antongiulio Faggiano, on behalf of NIKE Group

**Affiliations:** 1 Department of Clinical Medicine and Surgery, Federico II University, 80131 Naples, Italy; 2 Division of Endocrinology and Diabetes, Department of Internal Medicine I, University Hospital, University of Wuerzburg, 97080 Wuerzburg, Germany; 3 Department of Clinical Medicine, Bufalini Hospital, 47521 Cesena, Italy; 4 Clinica Medica 3, Department of Medicine, DIMED, University of Padova, 35128 Padova, Italy; 5 Department of Diagnostics and Public Health, Section of Pathology, University and Hospital Trust of Verona, 37126 Verona, Italy; 6 UOC of Oncology, AO dei Colli, Monaldi Unit, 80131 Naples, Italy; 7 Department of Experimental Medicine, Sapienza University of Rome, 00161 Rome, Italy

**Keywords:** neuroendocrine neoplasms, bone metastases, bone microenvironment, skeletal-related events, epithelial-to-mesenchymal transition, microRNA, prognosis, treatment, denosumab

## Abstract

Bone represents a common site of metastases for several solid tumors. However, the ability of neuroendocrine neoplasms (NENs) to localize to bone has always been considered a rare and late event. Thanks to the improvement of therapeutic options, which results in longer survival, and of imaging techniques, particularly after the introduction of positron emission tomography (PET) with gallium peptides, the diagnosis of bone metastases (BMs) in NENs is increasing. The onset of BMs can be associated with severe skeletal complications that impair the patient’s quality of life. Moreover, BMs negatively affect the prognosis of NEN patients, bringing out the lack of curative treatment options for advanced NENs. The current knowledge on BMs in gastro-entero-pancreatic (GEP) and bronchopulmonary (BP) NENs is still scant and is derived from a few retrospective studies and case reports. This review aims to perform a critical analysis of the evidence regarding the role of BMs in GEP- and BP-NENs, focusing on the molecular mechanisms underlining the development of BMs, as well as clinical presentation, diagnosis, and treatment of BMs, in an attempt to provide suggestions that can be used in clinical practice.

## 1. Introduction

Neuroendocrine neoplasms (NENs) are a heterogeneous group of tumors arising from cells with a neuroendocrine phenotype, which originate most frequently from the gastro-entero-pancreatic (GEP) and the bronchopulmonary (BP) tract [1,2]. Although NENs traditionally were considered a rare cancer, in the last few decades, there has been a rapid increase in the incidence due to more extensive use of modern imaging techniques [3,4]. The first symptoms of the disease are often related to tumor burden or to specific syndromes caused by hormones and neuroamines secretion in functioning tumors. Nevertheless, the incidental diagnosis of NEN is becoming more frequent. NENs could present a different aggressive behavior varying from slow-growing tumors, which represent the majority of cases, to high aggressive carcinomas (NEC) [1,2,5].

Despite the slow progression, a large proportion of patients present with distant metastases at diagnosis, mostly localized in the liver [4,6,7]. Bone metastases (BMs) are usually considered a late event in NEN patients, often occurring after the development of liver metastases, and frequently remain undetected [6]. Thus, BMs in NENs are considered extremely rare and lacking clinical relevance. However, similar to what has happened for NENs as a whole, an increased incidence of BMs have been reported more recently compared with previous studies [8,9,10,11,12,13,14]. The ability of NENs to metastasize to the bone represents a recent awareness, most likely due to improving imaging techniques and longer survival than to a real change in biological behavior.

Nowadays, data from the literature on BMs in NENs are still scant, and comprehensive evaluations of this topic are lacking. This review aims to perform a critical analysis of the literature, reporting the evidence on incidence, clinical presentation, pathogenesis, diagnosis, prognostic implication, and treatment of BMs in GEP- and BP-NENs.

## 2. Methods

Extensive research of the international literature included in the PubMed database was conducted up through August 2019. The following keywords were used: neuroendocrine neoplasms, gastro-entero-pancreatic neuroendocrine tumor, lung neuroendocrine tumor, carcinoid, bone metastases, epidemiology, molecular pathway, epithelial-to-mesenchymal transition, microRNA, symptoms, skeletal-related event, imaging, computed tomography, magnetic resonance imaging, bone scintigraphy, positron emission tomography (PET), biomarkers, prognosis, treatment, radiotherapy, Peptide Receptor Radionuclide Therapy (PRRT), bisphosphonate, and denosumab. Boolean operators were also used to improve the quality of the search. Titles and abstracts of the articles were first screened to identify the most relevant papers, and references included in the selected studies were taken into consideration. Articles written in languages other than English, letters to editors, and abstracts to conferences were excluded. The authors screened and discussed all the selected studies before including them in this review.

## 3. Epidemiology of BMs in GEP- and BP-NENs

The epidemiological data on BMs in NENs are derived only from retrospective studies, and even though the incidence is recently increasing, it still remains lower than those reported in an autoptic study [15], indicating that the real incidence of BMs is underestimated.

The most extensive data on BMs epidemiology in NENs are derived from the Swedish Cancer Registry by Riihimaki et al., which contains information on the incidence of metastases in 7334 patients with NENs [9]. Among this large cohort of patients, 1842 (25.1%) patients presented with metastases, predominantly in the liver, followed by other intra-abdominal sites and bone. Notably, BMs have been reported in 4.1% of all patients, representing 16.4% of all metastases [9]. Patients with pancreatic, small intestine, and BP-NENs had a higher risk of developing BMs compared with those with other primary tumor sites. The authors also reported a lower incidence in women than men, speculating that this discrepancy could be due to the protecting role of estrogens, which inhibit bone resorption by inducing osteoclast apoptosis and stimulate the production of osteoprotegerins [9]. A similar frequency of BMs was also reported by the Spanish National Cancer Registry and by a French retrospective multicenter study (5% and 6.4%, respectively) [7,16].

A more recent single-center German study by Scharf et al. reported BMs in 12.6% of 677 patients with NENs documented at the initial diagnosis (synchronous metastases, 35.3% of cases) or during the follow-up (metachronous metastases, 64.7%) [10]. The median age at the diagnosis of BMs was 57.3 years (range 24.4–79.7), and the majority of patients (83.5%) had well and moderately differentiated NENs with grading of G1/G2. Similar to the Swedish Registry, the most common primary tumor sites were the small intestine and pancreas (37.6% and 30.6% of cases, respectively) [10]. Similar results were also reported by another study conducted at Ohio State University Medical Center in 341 patients with well-to-moderate differentiated NENs [11]. BMs were found in 40 (11%) patients, among which 11 (27%) presented with synchronous and 29 (73%) had metachronous BMs. Different from the previous studies, the primary tumor size was significantly larger in patients with BMs compared to those matched for sex, age, and primary tumor site with advanced tumor stage but without BMs [11]. In a second study by Van Loon et al., also including another tertiary United States academic center in addition to that mentioned above, BMs were reported in 9% of carcinoids, including well-differentiated non-pancreatic GEP-NENs, and 8% of pancreatic NENs [12]. In this cohort, patients with BMs were younger (49 vs. 54 years old) and had more frequently associated liver metastases compared with those without [12]. In a recent Italian single-center retrospective analysis investigating a large number of BP-NEN patients (*n* = 348), Peri et al. reported BMs in 12% of all cases [14]. Atypical carcinoid represented 46.3% of all metastatic BP-NENs [14]. Bone represented the second site of metastases (42% of cases) after the liver in patients with metastatic lung NEN [17]. All these results were confirmed by a very recent multicenter study by Alexandraki et al., which found the pancreas and the small intestine to be the most common primary tumor sites (30% and 27%, respectively). Moreover, the majority of patients with BMs presented with associated liver metastases [18]. Different from the Swedish Cancer Registry [9], none of the abovementioned studies found significant differences in BMs frequency according to gender, although bone lesions were slightly more frequent in male patients [10,11,12].

In conclusion, BMs were reported in 4–12% of patients, representing the third site of metastases in NEN patients. The most common primary tumor sites were pancreatic, small intestine, and lung NENs [7,9,10,11,12,13,14,16,18]. However, the natural history of BM is still disputed, and data regarding the development of synchronous or metachronous metastases are contradictory [11,14,18]. Thus, prospective studies are urgently needed to better evaluate the incidence and the natural history of BMs.

## 4. Molecular Pathways of BM Development

The development of BMs is a multistage process characterized by dynamic crosstalk between tumor cells and bone [19]. Tumor cells, before escaping from the primary site, release cytokines, exosomes, and growth factors that disrupt bone microenvironment, causing the formation of a “pre-metastatic niche” [20]. After acquiring an invasive phenotype, tumor cells enter into the circulation and colonize the distant tissues, where they previously prepared the pre-metastatic niche, establishing a “metastatic niche”. The invasive phenotype seems to be enhanced by the epithelial-to-mesenchymal transition (EMT), which allows the epithelial tumor cells to acquire a motile mesenchymal phenotype [21]. Thus, EMT plays a crucial role in metastasis development and, as well as growth factors and cytokines involved in this process, also stimulates the formation of BMs [22].

**Figure 1:**
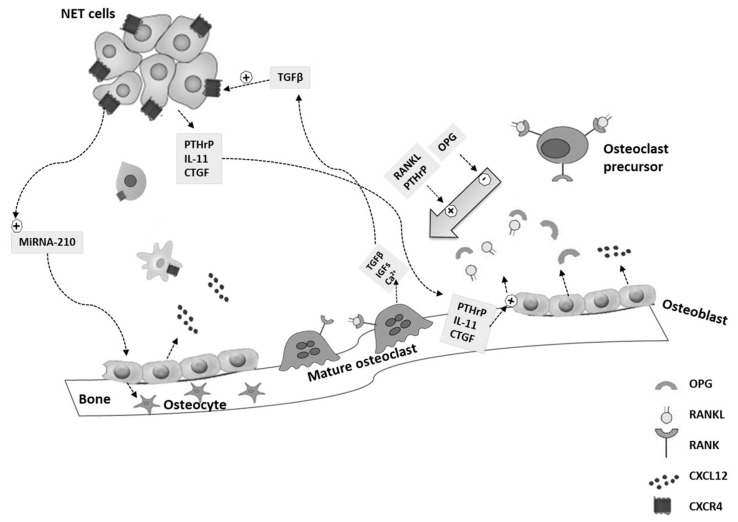
Molecular mechanism of bone metastasis in neuroendocrine neoplasms: the “vicious cycle”. Neuroendocrine neoplasms (NENs) cells secrete pro-osteolytic factors, including the parathyroid hormone-related protein (PTHrP), interleukin-11 (IL-11), and the connective tissue growth factor (CTGF), which stimulate the activator receptor of the nuclear factor-kappa B ligand (RANKL) production by osteoblasts and/or the decrease of osteoprotegerin (OPG) within the bone stroma. Thus, RANKL induces osteoclast formation. Osteoclastic bone resorption causes the release and activation of growth factors, including the transforming growth factor-β (TGFβ), the insulin-like growth factors 1 (IGF1), and calcium ions (Ca2+). TGFβ can increase tumor production of the C-X-C motif receptor 4 (CXCR4) in NEN cells. The C-X-C motif chemokine-ligand-12 (CXCL12) is mainly produced by osteoblasts and can attract CXCR4-overexpressing NEN cells. CXCR4 and CTGF play a role in the migration of NEN cells to the bone. Micro-RNA (miRNA)-210 is upregulated in NEN cells and regulates the differentiation of osteoblasts into osteocytes.

Current evidence suggests that cancer cells can remain occult in a dormant state for decades in the metastatic niche before proliferating and forming metastases [23,24]. Molecular mechanisms behind the escape from dormancy are largely unknown and maybe influenced by several factors, including changes in bone microenvironment and osteoclast activation [20,25]. A unique feature of BMs is that tumor cells are not able to destroy the bone directly, but they need to stimulate osteoclasts to degrade the bone extracellular matrix (ECM) [26]. This crosstalk between tumor cells and bone microenvironment promotes a “vicious cycle” (Figure 1) [27]. When tumor cells escape from the dormant state, they start to proliferate and secrete several factors, including the connective tissue growth factor (CTGF), interleukin-11 (IL-11), prostaglandin E (PGE2), and parathyroid hormone-related protein (PTHrP). All these factors cause the increase of the receptor activator of nuclear factor-kappa B (RANK) ligand (RANKL) and/or the decrease of its inhibitor osteoprotegerin (OPG) within the bone stroma [26,28]. RANKL is a member of the tumor necrosis factor (TNF) ligand superfamily and is expressed by bone stromal cells of the osteoblast lineage. After binding its receptor RANK, RANKL mediates the differentiation and activation of osteoclasts. Activated osteoclasts secrete cathepsin K and other proteinases into the bone matrix, which degrade type I collagen, resulting in the degradation of ECM and bone remodeling, with consequent formation of BMs (Figure 1) [26,28].

The impairment of the RANKL/OPG pathway is frequently observed in different tumor types, including GEP- and BP-NENs [29]. The subsequent bone resorption causes the release of calcium and bone-derived growth factors from bone ECM, including the transforming growth factor-β (TGFβ), the insulin-like growth factors 1 (IGF1), and the platelet-derived growth factor (PDGF). It also increases the secretion of growth and angiogenic factors by the osteoblast, including the C-X-C motif chemokine-ligand-12 (CXCL12), which stimulates tumor cell proliferation both directly and indirectly. Moreover, physical properties of the bone ECM, including hypoxia, acidic pH, and high extracellular calcium levels, create an environment favorable for tumor growth that, together with the abovementioned growth factors, stimulate tumor cell homing and proliferation and bone destruction, creating the feed-forward loop known as the “vicious cycle” (Figure 1) [26,27,28,30]. Tumor-derived TGFβ is central to this cycle, because it stimulates cancer cells to produce additional PTHrP, which mediates the production of RANKL by osteoblasts, leading to a perpetuation of the vicious cycle [27,31,32] (Figure 1). Furthermore, the RANKL/OPG pathway is also involved in EMT promotion [33].

The understanding of the vicious cycle leads to the identification of molecular targets that could be useful for the development of drug strategies to arrest tumor progression to bone. Due to its central role in BM development, RANKL is one of the most important targets for the treatment of BMs. Denosumab is an anti-RANKL antibody that blocks osteoclast formation by inhibiting the RANKL–RANK interaction, mimicking the physiological role of OPG [34]. However, the production of other endogenous and tumor-derived factors in the metastatic niche, including cathepsin K, IL-8, and vascular endothelial growth factor (VEGF), contributes to osteoclast bone resorption independently of RANKL [25,35]. These escaping mechanisms from RANKL activation contribute to the failure of RANKL inhibitors treatment [31]. Cathepsin K, mostly expressed by osteoclasts, represents another interesting target for BM therapy. However, although results from in vitro and preclinical studies have been promising, clinical trials evaluating cathepsin K inhibitors have been discontinued due to profound side effects [36,37].

CXCL12, together with its C-X-C motif receptor 4 (CXCR4), which is overexpressed in malignant cells, plays an important role in the development of BMs, because its activation promotes tumor cell proliferation and angiogenesis (Figure 1) [38]. Circelli et al. first demonstrated an impaired expression of the CXCR4/CXCL12 pathway both in vitro and in NEN tissues [39]. The authors showed high CXCR4 mRNA levels in NCI-H727 (bronchial-NEN) and BON (pancreatic-NEN) cell lines, and overexpression of both *CXCL12* and *CXCR4* in GEP-NENs (n = 36) compared with normal tissues [39]. These results were confirmed by another in vitro study by Cives et al. on three different pancreatic NENs cell lines, the BON1, CM, and QGP1 [40]. These cell lines showed a high expression of CXCR4 and low secretion of CXCL12, resulting in a CXCR4^high^/CXCL12^low^ profile. The authors demonstrated that CXCR4 stimulation increased cell osteotropism and drove cells to EMT-like transcriptional shift in vitro. The EMT process was not observed in cell lines with a CXCR4^low^/CXCL12^high^ profile, including H727 lung and CNDT 2.5 midgut carcinoid cells [40]. In another study by the same group, the authors investigated the critical role played by the EMT process in the development of BMs in NENs [41]. They evaluated the expression of eight different EMT-related factors by immunohistochemistry in 44 NEN tissues. Among these factors, they found that the overexpression of CXCR4 and CTGF, associated with a low expression of TGFβ1, significantly correlated with an increased risk of BMs, suggesting an implication of the EMT in NEN osteotropism. Particularly, by combining the staining score of the three proteins, the authors were able to identify NEN patients with a sensitivity and specificity of 91% and 100%, respectively [41]. A very recent study by Rizzo et al., demonstrated that the presence of circulating tumor cells (CTCs) was significantly associated with BMs in a large cohort of 254 patients with GEP-NENs and that the proportion of CXCR4-positive CTCs was slightly higher in patients with BMs compared with those without (56% vs. 35%, *p* = 0.18) [42]. All these studies suggested that CXCR4 plays an important role in the development of BMs in NEN patients and confirmed that NENs are capable of initiating the EMT process [43,44,45,46], which could be stimulated by CXCR4 [39]. A limited number of pre-clinical studies have demonstrated that the inhibition of the CXCR4/CXCL12 pathway using CXCR4 antagonists, including Plerixafor and CTCE-9908, reduced bone metastatic burden in breast and prostate cancer models [47,48,49]. However, in further in vivo models of prostate cancer, these promising results were not confirmed [31,50]. Therefore, it remains unclear whether targeting this pathway could be useful to inhibit the development of BMs.

Several preclinical studies have identified microRNAs (miRNAs) as important regulators of the metastases process and osteotropism [51,52,53]. Two different studies reported that miRNA-21 was upregulated in pancreatic and BP-NENs and was associated with liver and lymph node metastases [54,55]. Interestingly, miRNA-21 played a key role in osteoblast differentiation, stimulated the expression of different matrix metallopeptidases (MMP), including MMP2, MMP9, and MMP13, and promoted the EMT process [56]. Moreover, miRNA-155 has been shown to be upregulated in high-grade lung NENs compared with both typical and atypical carcinoids [54]. This miRNA inhibited the TGFβ pathway, modulating osteogenic differentiation, and was associated with metastatic spread in breast cancer [57]. Finally, miRNA-210 has been reported to be upregulated in GEP-NENs and associated with metastatic spread [53,58]. It has been said that miRNA-210 stimulates the expression of vascular endothelial growth factors and enhances the differentiation rate of bone marrow mesenchymal stem cells into osteoblasts [59]. Due to these findings, the role of miRNAs in the pathogenesis and development of BMs in NENs should be better investigated in future studies.

## 5. Clinical Presentation of BMs and Skeletal-Related Events

In NEN patients, BMs are the third most common site of metastasis after the liver and other intra-abdominal sites and are frequently associated with liver metastases [9]. The most frequent sites of BMs in NEN patients are the axial skeleton, mostly at vertebra levels, followed by the pelvic region and ribs, whereas only a small percentage of patients have BMs at the appendicular skeleton, which are often associated with axial BMs [10,11,18,41]. Moreover, BMs occur more frequently as multiple lesions and only in a small percentage of cases as a single metastasis [18].

In 20%–40% of cases, BMs are asymptomatic and are detected during tumor staging and follow-up [12,41], whereas only a small percentage of cases are diagnosed after developing serious complications [18,60]. Therefore, the management of BMs was considered only as a palliative treatment for a long time. However, skeletal complications associated with BMs, termed as skeletal-related events (SREs), which include bone pain, pathological bone fractures, spinal cord compression, and hypercalcemia [61], are often accompanied by morbidity that could cause impairment in activities of daily living and worsen patients’ quality of life (QoL) [60]. In some cases, patients with SREs have required emergency intervention with a consequent high rate of hospitalization. Thus, SREs are related to a significant increase in medical costs with an impact on the health care system [62]. Finally, SREs also affect the prognosis of patients, because the deterioration of the general condition may cause the discontinuation of treatment, leading to tumor progression [61].

Overall, 59–77% of GEP- and BP-NENs patients with BMs were symptomatic [10,12,41]. None of the evaluated parameters, including tumor histology, tumor grade, gender, and radiological appearance of BMs, significantly correlated with the occurrence of SREs [12,41]. Different from the studies mentioned above, Kavecansky et al. reported a lower frequency of SREs (35%), because they evaluated only spinal cord compression and pathological fractures, without including bone pain [11]. In the recent study by Alexandraky et al., SREs were reported in only 9% of the patients [18]. This discrepancy could be due to the use of more advanced imaging procedures, which allowed an earlier diagnosis of BMs, preventing the development of SREs.

### 5.1. Pain

Several studies have shown bone pain to be the most common symptom, reported in 42.4%–100% of patients with metastatic GEP- and BP-NENs [10,12,13,41,63]. Bone pain could frequently be disproportionate to the degree of bone involvement and could impair performance status, affecting patients’ work, motility, and sleep [6]. Scharf et al. reported that 28% of patients with BMs complained about pain at the initial diagnosis, whereas another 14.1% of patients developed pain during the follow-up [10]. These results indicated that patients who were initially asymptomatic might develop pain, underlying the importance of early drug intervention to improve bone health and prevent SREs.

### 5.2. Pathological Fractures

Bones weakened from metastasis could break or fracture. Pathological fractures represent the primary cause of bone pain, impair the patients’ mobility and QoL, and are mostly treated with orthopedic surgery [60,64]. Between 4% and 15% of NEN patients with BMs develop pathological fractures that occur predominantly in the axial skeleton [11,12,41]. Scharf et al. showed that pathological fractures occurred in 7.1% of patients at the initial diagnosis, increasing to 11.8% during the follow-up, whereas 3.5% of cases presented a high risk for fracture lesions [10].

### 5.3. Spinal Cord Compression

Local infiltration by metastasis of the surrounding tissue beyond the cortical bone, as well as the displacement of bone fragments secondary to pathological fracture, could be associated with spinal cord compression [65]. Spinal cord compression has been considered to be an oncological emergency and, in the early stage, it has been associated with radicular pain that could progress to neurological signs and symptoms, including motor weakness, gait disorders, sensory deficits, and urinary, bowel, and sexual dysfunction [66]. Signs and symptoms related to spinal cord compression were reported in 9%–20% of patients with GEP- and BP-NENs [10,11,12,41] and mostly involved the thoracic and lumbar spine, although cervical involvement was also reported [67].

### 5.4. Hypercalcemia

Hypercalcemia was rarely described in NEN patients with BMs and has been reported in up to 3% of patients [10,41]. However, no GEP- and BP-NEN patients presented hypercalcemia due to BMs in the majority of studies [11,12,13].

## 6. Diagnosis of BMs

Different diagnostic tools, including morphological and functional imaging, tumor markers, and a careful evaluation of symptoms, are necessary for the diagnosis of BMs.

### 6.1. Imaging Procedures

The European Neuroendocrine Tumor Society (ENETS) Consensus Guidelines on NEN-related BMs, published in 2010, recommend the use of both anatomic imaging, i.e., magnetic resonance imaging (MRI) and functional whole-body imaging methods, including bone scintigraphy and somatostatin receptor scintigraphy (SRS), for the detection of BMs [6].

Before the introduction of PET with gallium (Ga) peptides, the initial diagnosis of BMs in NEN patients was made frequently by MRI or computed tomography (CT) [10,11,41]. Bone lesions deriving from NENs appeared predominantly osteoblastic (up to 83% of cases), whereas osteolytic or mixed patterns were less frequent [11,41]. MRI was shown to have a sensitivity of nearly 100% for the detection of bone marrow metastases [13,68], and particularly, whole-body MRI associated with body diffusion-weighted imaging (DWI) better distinguished benign from malignant bone lesions [69]. Recently, no significant differences between CT and MRI for the detection of BMs have been demonstrated [18]. Another recent study comparing whole-body DWI MRI and [11C]-5-hydroxytryptophan (5-HTP) PET-CT found a good concordance between these two methods for the detection of BMs [70].

Bone scintigraphy with the use of technetium (^99m^Tc) showed a sensitivity of 90%–100% for the detection of BMs and had a higher diagnostic performance than SRS [6,71], although a previous study showed contrary results [72]. However, bone scintigraphy presented limitations in the low spatial resolution and could not distinguish metastases from a repair process [73].

In the last few decades, the superior value of PET/CT over SRS in the diagnosis of NENs, particularly after the introduction of ^68^Ga-DOTA-peptides that specifically bind to somatostatin receptors (SSRs), has been widely demonstrated [74,75,76]. All three ^68^Ga-DOTA-peptides (DOTATOC, DOTANOC, and DOTATATE) specifically bind to SSR2 and SSR5 with different affinity, whereas only DOTA-NOC presents affinity for SSR3 [77]. Putzer et al. demonstrated in a cohort of 51 NEN patients referred for PRRT that ^68^Ga-DOTATOC PET/CT had a sensitivity of 97% and specificity of 92% for the detection of BMs, with only 2% false-positive and false-negative cases [73]. Moreover, they showed that ^68^Ga-DOTATOC PET/CT detected a significantly higher rate of BMs compared with CT (*p* < 0.001) [73]. A recent study investigating a larger cohort of NEN patients (n = 535) confirmed the high sensitivity of ^68^Ga-DOTATOC-PET/CT in detecting vertebral metastases [78]. Similar results were also obtained with the other ^68^Ga-DOTA-peptides. Albanus et al. reported a significant increase of sensitivity and specificity of ^68^Ga-DOTATATE-PET/CT compared with CT for the detection of BMs (100% vs. 47% for sensitivity and 89% vs. 49% for specificity, respectively) [79]. Ambrosini et al. showed the superiority of ^68^Ga-DOTANOC-PET/CT compared with CT for the evaluation of BMs, which presented a higher sensitivity (100% vs. 80%) and a comparable specificity (100% vs. 98%) in a cohort of 223 patients with NENs [80]. Particularly, the authors demonstrated that CT was able to detect bone lesions only in 79% of patients with positive ^68^Ga-DOTANOC-PET/CT, whereas the lesions were positive with both imaging methods in 60% of cases. Furthermore, the characteristics of the bone lesion in CT (sclerotic, lytic, mixed) did not change the PET results [80]. False-positive results with ^68^Ga-DOTA-peptides-PET/CT could occur in patients with angiomas, inflammatory processes, and lymphoma, due to the expression of SSRs on activated lymphocytes [81], whereas false-negative results could occur in those tumors with low or absent SSR expression, indicating the presence of dedifferentiated cells [82]. In these cases of poorly differentiated NENs, the use of PET/CT with the metabolic tracer fluorodeoxyglucose (^18^F-FDG) [82] or with fluoro-18-L-dihydroxyphenylalanine (^18^F-DOPA) could be preferred [83]. Overall, the increased sensitivity and specificity in the detection of BMs with PET/CT frequently leads to an upstaging of the disease, causing a modification of therapeutic course in 25%–60% of patients at the beginning of PRRT or chemotherapy [79,82,84,85].

**Figure 2:**
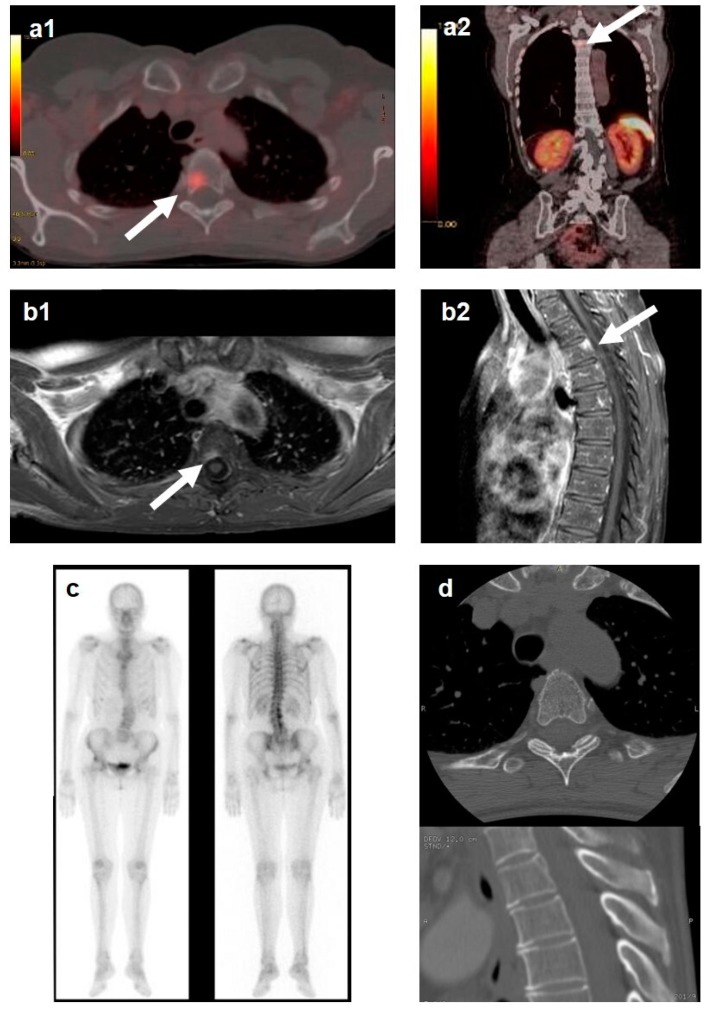
Thoracic vertebrae (T4) bone metastasis (BM) was diagnosed two years after a liver metastasis in a 66-year-old woman with a history of ileal neuroendocrine neoplasm. Somatostatin receptor scintigraphy (SRS) was performed twice in the last four years of follow-up and did not detect BM. (**a1**,**a2**) T4 vertebra BM was first detected by 68Ga-DOTATOC positron emission tomography (PET)/ computed tomography (CT); (**b1**,**b2**) BM was confirmed by magnetic resonance imaging (MRI); (**c**) 99mTc-bone scintigraphy did not detect BM; (**d**) computed tomography was also negative for BM. Arrow indicates the BM.

To better underline the differences among the imaging procedures, we reported a case of a 66-year-old woman with a history of ileal NEN and a metachronous T4 vertebral metastasis that was first detected by 68Ga-DOTATOC PET/CT (Figure 2a1,a2) and confirmed by MRI (Figure 2b1,b2). The same metastasis was not detected with the previous ^99m^Tc-bone scintigraphy (Figure 2c) and CT scan (Figure 2d).

### 6.2. Biomarkers

Bone remodeling associated with BMs may cause the release of specific bone turnover biomarkers (BTMs), which are measured in blood and/or urine [86]. In NEN patients with suspected BMs, the ENETS Guidelines suggest the measurement of BTMs, including serum bone-specific alkaline phosphatase (BSAP), procollagen type I (PI) N-terminal propeptide (PINP), and C-terminal propeptide (PICP) as markers of bone formation and C- and N- telopeptide of type I collagen (CTX and NTX, respectively) as markers of bone resorption. BTMs should be evaluated in addition to general markers of NENs, including chromogranin A (CgA), as well as specific hormones and peptides related to functional tumors [6].

In a first study comparing NEN patients with or without BMs, matched for sex, age, and primary tumor site, serum levels of BSAP, PINP, and NTX were not different between the two groups and did not correlate with urinary 5-hydroxyindoleacetic acid (5-HIAA), age, type of treatment, and time of the diagnosis [13]. No differences in tumor marker levels, including gastrin, pancreatic polypeptide, glucagon, and pancreastatin, were detected in NEN patients with BMs as compared with patients with other types of metastases [11]. The RANKL/OPG pathway, considered to be a means to estimate the osteolysis rate, was evaluated in GEP- and BP-NENs by Milone et al. [29]. The authors showed that patients with BMs had decreased serum levels of OPG compared with those without BMs, speculating that the RANKL/OPG ratio might predict an early development of BMs [29].

However, BTMs seemed to be less influenced than expected in NENs. One possible explanation could be that NENs presented a slow growth in the majority of cases, having a wake impact on the bone metabolic processes. Moreover, patient’s characteristics, the presence of concomitant liver metastasis, and the treatment with somatostatin analogs (SSA), might influence BTM levels [6,86]. For all these reasons, BTMs could be not significantly altered in NEN patients, and except for the RANKL/OPG ratio, they did not appear to be useful in distinguishing patients with or without BMs.

## 7. Impact of BMs on Prognosis

The presence of BMs seemed to impact the prognosis of NEN patients negatively. However, it is difficult to evaluate the direct prognostic impact of BMs in these types of neoplasm, due to the incidence and the heterogeneity of NENs, as well as the frequent coexistence of multiple distant metastases.

To the best of our knowledge, only retrospective studies and one systematic review have analyzed this topic. In the study by Strosberg et al., evaluating 146 cases of metastatic midgut NENs, BMs represented a negative prognostic factor, because patients with BMs (*n* = 35) had a median survival of 32 months (95% CI 28–35 months) and a 5-year survival rate of 20% [87]. Subsequent studies confirmed these data, comparing overall survival (OS) of NEN patients with BMs and with other metastatic distant sites. The study by Scharf et al. demonstrated a significantly lower OS in patients with BMs than in those with other distant metastases (49.0 vs. 100.8 months; *p* = 0.01) [10]. In particular, synchronous BMs was associated with a poorer outcome compared with metachronous BMs [10]. Similarly, a study from Ohio State University Medical Center found that patients with BMs had a shorter OS compared with patients with metastatic disease but without skeletal involvement (median OS 52 vs. 98 months; *p* = 0.024) [11]. A difference between the histological subtype of NENs was reported by Van Loon et al. [12]. In patients with NENs metastasized to liver and bone, the median OS was 47.8 months, compared with 99.5 months in patients with liver metastases without BMs (*p* < 0.001). These data were not confirmed in high-grade NECs, whereas a similar trend was found in patients with metastatic pancreatic NENs, even if it did not reach statistical significance [12]. Peri et al. showed that BMs, together with liver metastases, age, Ki-67 index, and shorter time to recurrence significantly correlated with poorer prognosis both at univariate and multivariate analyses in BR-NETs [14]. The authors did not observe significant prognostic differences in terms of histological subtype (typical and atypical carcinoid) or between synchronous and metachronous metastatic disease even though patients with synchronous disease had significantly more metastatic sites [14]. The impact of synchronous and metachronous BMs was also analyzed in a systematic review, describing patients with BMs from NENs in a population of 152 patients from 129 papers [41]. Patients with synchronous BMs had a significantly shorter OS (12 months; 95% CI 6–20) in comparison to patients with metachronous BMs (36 months; 95% CI 24–48; *p* = 0.0002). At univariate analysis, synchronous bone involvement was the only predictor of poor prognosis (*p* = 0.0002) in patients with metastatic NENs, while metachronous BMs, type and tumor site, symptoms at presentation, and SREs occurrence appeared not to influence the outcome [41]. These results were recently confirmed by Alexandraki et al., who reported a shorter OS in patients with synchronous BMs compared with those with metachronous BMs (median OS 51.7 vs. 116.4 months; *p* < 0.001) [18].

The presence of BMs was also found to be one of the covariates associated with progression-free survival (PFS) in patients with advanced, well-differentiated NEN treated with SSA. Particularly, BMs were associated with a worse PFS, having a time ratio of 0.63 (95% CI 0.43–0.90) in the accelerated failure time (AFT) model to predict PFS [88].

All these studies demonstrated that the presence of BMs, and particularly synchronous osseous involvement, might negatively influence the clinical course and the prognosis of NEN patients.

## 8. Therapy of BMs

Current ENETS Guidelines concerning the management of BMs in NENs suggest bisphosphonates, administered either orally or intravenously, as the first therapeutic option [6]. Moreover, radiotherapy in the case of pain and adequate hydration in the case of hypercalcemia have been recommended [6]. RANKL inhibitor therapy, denosumab, was introduced in 2010; therefore, this therapeutic option was not considered in the guidelines as mentioned earlier. More recently, Farooki published the recommendations of the National Comprehensive Cancer Network Bone Health Task Force, also focusing on metastatic bone disease in solid tumors [89]. The author reported that bisphosphonates and denosumab could prevent SREs and relieve pain in patients with advanced cancer and BMs. However, the effects of antiresorptive agents on disease outcomes remain controversial [89]. External beam radiation therapy (EBRT) and/or surgery are often required both to prevent and to treat SREs [64,90,91]. A recent study showed that EBRT was able to relieve bone pain in 90% of treated BMs in NEN patients, independent from single-fraction vs. fractionated regimens, primary tumor site, chemotherapy received during RT, or radiation site [63]. In addition, among loco-regional therapies, thermal ablation techniques (radiofrequency-ablation/cryotherapy) must be mentioned. Studies conducted on other solid tumor types have showed that, especially cryoablation, could be used not only with a palliative intent but also with a curative intent in oligometastatic and/or metachronous diseases, with small-size (<2 cm) BMs and no cortical erosion [92,93]. Relative to this topic, an ongoing clinical trial (NCT03986593) is evaluating the clinical response and safety of cryoablation in bone metastases from thyroid, adrenal, and neuroendocrine tumors (Table 1).

In general, evidence from the literature about the management of BMs in NEN patients is scarce. At this time, our knowledge comes from studies conducted on other solid tumor types or retrospective studies on NENs. Furthermore, ongoing clinical trials are few and are not specifically focused on BMs in NENs (Table 1). However, interesting results could also come up from studies that do not have as the primary endpoint the response of BMs to a specific intervention (NCT02743741, NCT02489604, NCT03478358; Table 1). Long-time retrospective studies on NEN patients are in line with current guidelines. In the study by Kavecansky et al. on 40 patients, EBRT was given to symptomatic BMs in 22 (55%) patients, and 12 (30%) patients were treated with bisphosphonates [11]. The systematic review from Cives et al. showed that EBRT (38%) and surgery (25%) were the most frequently reported treatments for BMs. Only 3% of patients received bisphosphonates, whereas one-third of patients did not receive any form of bone-directed therapy [41].

Contrary to these studies, recent studies demonstrated a more frequent use of bone-directed therapy, also including denosumab. In the study by Van Loon et al., 67 (82%) patients with BMs received at least one form of bone-directed therapy: 50% received EBRT, 45% received bisphosphonate, 18% underwent surgical resection, 13% received treatment with ^131^I-metaiodobenzylguanidine (^131^I-MIBG), and 5% received denosumab. Moreover, 46% of patients were treated with more than one treatment modality [12]. In the study by Scharf et al., the most common treatments used were bisphosphonates (64.7%), followed by palliative radiation therapy (25.9%). Only three patients received surgical therapy (3.5%), and six patients received denosumab (7.1%) [10].

Although bone-directed therapy is routinely used for other tumor types with BMs, it has still not become part of the common clinical practice with respect to NENs. Probably, this form of resistance to treat BMs can be explained by the apparent absence of survival benefit in NEN patients treated with bisphosphonates. Kavecansky et al. did not show any statistically significant difference in terms of survival between patients treated or not treated with bisphosphonates (mean OS 52 vs. 32.5 months, respectively) [11]. Conversely, Scharf et al., examining the effects on survival of all bone-specific treatment, observed a trend for a longer median OS in patients who underwent bone-specific therapy compared with those without specific treatment [10]. Another very recent study demonstrated that the outcome of bone-related therapy independently predicted mortality. Particularly, NEN patients with a response or stable disease after bone-specific therapy had better OS and BMs-related survival compared with non-responding patients [18]. In a small subgroup of patients, the authors also evaluated different schemas of bisphosphonate treatment (monthly vs. other schemes), reporting no significant difference among them [18]. This result confirmed what was already reported in a large, randomized, open-label clinical trial (NCT00869206) with respect to other cancer types, demonstrating that the use of the monthly standard dosing interval of zoledronic acid (ZA), the only approved bisphosphonate in NENs, compared with the three-month interval did not result in an increased risk of SREs over two years [94]. A large randomized phase III trial (NCT00330759) and a later post hoc analysis showed that denosumab was slightly more efficacious than ZA in preventing or delaying the risk of onset of first and subsequent SREs in patients with advanced cancer metastasized to bone, including NENs [95,96]. However, the incidence of hypocalcemia in patients treated with denosumab was higher than with ZA treatment (12.4% vs. 5.3%, respectively) [97]. Since the natural history of NENs is characterized mostly by long OS, the impact of the bone-directed therapies should be investigated on QoL rather than OS. For this reason, in patients with relatively indolent progression, three-month ZA should be recommended rather than the monthly schema, because the three-month dosing interval is easiest for the patient, likely results in fewer side-effects, and is cheapest for the health care system, whereas denosumab should be recommended for very aggressive diseases [98].

Other potential perspectives could come from ongoing clinical trials on new molecular target therapies in advanced metastatic cancers (NCT00004074, NCT00005842; Table 1), even if the primary endpoint of these studies is the evaluation of the maximum tolerated dose of the investigated drugs. Promising results may arise from several ongoing trials that are evaluating the efficacy of different radiopharmaceuticals on BM treatment in solid tumors, although the “neuroendocrine milieu” is not considered. Among these radiopharmaceutical, radium-223 dichloride (radium-223), a new radioactive agent approved in castration-resistant prostate cancer, is the most interesting [99]. Radium-223 is a calcium mimetic that forms hydroxyapatite complexes in areas of high osteoblast activity and increased bone turnover, binding particularly osteoblastic BMs [100]. Moreover, it releases energy in alpha particles, which penetrates only cells over a short range (<1 mm), leading to lower bone marrow toxicities compared with other radiopharmaceuticals [101]. Because BMs in NEN are predominantly osteoblastic, they could be a good target for radium-223 treatment. Only a phase I study evaluating the safety of radium-223 associated with weekly paclitaxel included one single NEN patient [102]. Thus, clinical studies evaluating the efficacy of radium-223 in NEN patients are urgently needed.

Nowadays, many questions related to the management of BMs from NENs remain without answers, and predictive factors of response for bone-directed treatments are urgently needed to identify and to find the correct timepoint for starting these treatments. In clinical practice, BM therapy is often not suggested until the onset of bone pain or the development of other SREs, maybe nullifying its potential protective effect. Potential side effects, such as atypical femur fracture, hypocalcemia, renal complications, and osteonecrosis of the jaw, may limit or delay ZA and denosumab use [89]. The risk of developing these effects should be considered before starting therapy. Further prospective interventional studies are needed to clarify the potential effects of bone-directed therapy in delaying or preventing the onset of SREs in NEN patients. Currently, the choice of bone directed therapy, as well as its frequency and duration, should be tailored to the patient considering each case individually.

## 9. Impact of BMs on NEN Treatment

The presence of BMs seems to affect the clinical course and prognosis of NEN patients, but its role in defining the therapeutic strategies of these tumors has still not been clarified. BMs are usually accompanied by metastases at other distant sites, making it difficult to understand if metastatic bone involvement should indicate a specific treatment or if it should modify the therapeutic algorithm of NENs. ENETS Guidelines consider multiple treatment options in metastatic disease, but the appropriate therapeutic decision should always be discussed within a multidisciplinary tumor board [103].

As reported in current Guidelines regarding the management of BMs in NEN patients, the influence of surgery on survival in patients with BMs has not been formally studied. Excision could be evaluated in cases of solitary lesions at a single organ site [6]. The presence of BMs indicates an advanced tumor stage, in which a systemic therapy should be recommended, including SSA, alpha-interferon (IFN), chemotherapy, the mTOR inhibitor everolimus, the tyrosine kinase inhibitor (TKI) sunitinib, and PRRT [104].

Among these systemic therapies, some studies highlight the potential advantages of PRRT in the treatment of BMs. For a long time, ^131^I-MIBG therapy has been considered in the management of metastatic NENs, especially for its palliative efficacy. Available data reveal that ^131^I-MIBG could determine symptomatic benefit in more than 50% of patients, but its results, in terms of efficacy, are not equally satisfying [6]. Subsequently, new SSA labeled with the β-emitting radionuclides ^177^Lutetium and ^90^Yttrium were introduced. A prospective study showed a favorable response of ^177^Lu-octreotate in terms of survival in 310 GEP-NEN patients, noticing that time to progression was significantly shorter in patients having BMs. Nevertheless, the authors did not attribute this poorer response to the scarce effectiveness of PRRT in patients with BMs [105]. Furthermore, two retrospective studies on a small cohort of GEP-NEN patients with BMs demonstrated an optimal disease control rate after PRRT with ^177^Lu-octreotate, as well as an improvement of bone pain in symptomatic patients [106,107]. Significant hematological toxicity was reported in a non-negligible percentage of patients and was reversible within 22 months [106,107]. Only in the case of impaired hematological function, i.e., Hb <5 mmol/L (8 g/dL), platelets <75 × 109/L, leukocyte count <2 × 109/L, was PRRT contraindicated [108].

Regarding the use of chemotherapeutic agents, such as 5-fluorouracil (5-FU) and IFN, it is often limited from the presence of high load BMs, which could be related to a limited bone marrow reserve and, consequently, to a high risk of hematological toxicity [109]. Among the chemotherapies used in NEN patients, only everolimus and TKI showed a potential effect on BMs in other solid tumors. A study in an osteotropic breast cancer model demonstrated that everolimus had a bone-protective efficacy both in vitro and in vivo [110]. In a recent clinical trial on patients with advanced renal cell carcinoma and bone metastases, the TKI cabozantinib has been associated with an improvement of survival parameters when compared with everolimus [111].

Nowadays, PRRT with radiolabeled SSA seems to represent the most promising treatment in the case of BMs in NEN, but further prospective studies are needed to identify potential molecular targets that could be used for BM treatment. Three clinical trials on PRRT with the potential ability to solve some doubts are actually ongoing, even though the primary outcomes of these studies are not focused on bone (Table 1).

## 10. Conclusions

The ability of NENs to localize in bone has always been considered to be a rare and late event of its natural history and peculiar to some NEN types [6]. During the last decade, thanks to the improvement of therapeutic options, which prolongs the survival of NEN patients, together with the improvement of imaging techniques, particularly after the introduction of ^68^Ga-PET/CT, the diagnosis of BMs in NENs is increasing, reaching 12% of cases [9,10,11,12,13,14]. Current retrospective studies might have underestimated BMs epidemiology in NENs, as they mostly referred to previous and less sensitive imaging tools. Thus, the epidemiology of BMs in NENs is still to be defined. Considering the group of GEP- and BP-NENs, patients with small intestine, pancreatic, or lung NENs have been shown to have a greater risk of BMs compared with other primary tumor sites [7,9,10,11,12,13,14,16,18]. The importance of BMs is also suggested by in vitro studies, which underline that NEN cells exhibit osteotropism [23,112] and that the mechanisms involved in the formation of BMs, including the EMT and the alteration of RANKL/OPG and CXCR4/CXCL12 pathway, are similar to those found in other tumor types [39,40,41]. However, the validation of biomarkers that could be used to accurately predict the risk of BMs in NEN is a critical point that still remains. Moreover, it is urgently needed to find new potential molecular markers that could be used for targeted-drug development strategies.

In GEP- and BP-NEN patients, BMs frequently occur as multiple lesions in the axial skeleton, are associated with liver metastases, and are predominantly osteoblastic [10,11,41]. Despite the fact that BMs could be associated with SREs, can worsen patients’ QoL [60], and can negatively affect prognosis [10,11,12,14,41,87], the management of these metastases in NENs is still under debate. Moreover, because the ENETS Guidelines are from 2010, they do not consider the use of the newest targeted bone therapy, denosumab [6]. Starting from evidence deriving from other solid tumor types, bone-targeted agents, including bisphosphonates (i.e., ZA) and RANKL inhibitor (denosumab), together with EBRT, should be used for the treatment of BMs to prevent SREs [10,12] and improve patient prognosis [18]. In addition, other loco-regional therapies, and particularly cryotherapy, could represent a promising therapeutic option [92,93]. Because the presence of BMs indicates an advanced tumor stage, the association with systemic therapies, including SSA, IFN, chemotherapy, everolimus, sunitinib, and PRRT should be recommended [106,107]. The analysis of the available literature does not allow for the drawing of conclusions about the impact of BMs on therapeutic choice in NENs. The future directions that come from ongoing studies will identify new pathways and allow promising drugs, such as Radium-223, to be explored. For the moment, due to the complexity of these patients, an appropriate therapeutic decision should always be discussed within a multidisciplinary tumor board [6,103].

In conclusion, further in vitro and prospective studies are urgently needed to understand better the tumor biology of BMs, to detect high-risk patients at an earlier stage, and to evaluate the best strategy to prevent SREs and treat BMs.

## Figures and Tables

**Table 1:** Ongoing clinical trials for the treatment of bone metastases in neuroendocrine neoplasms.

ID	STATUS ^1^	PHASE	STUDY TITLE	INTERVENTION	PRIMARY OUTCOME
NCT03986593	Recruiting	Not Applicable	Cryoablation of Bone Metastases from Endocrine Tumors	Cryoablation	Change in the local disease status of the cryoablation-treated bone metastases; absence of neurological impairment and/or pain.
NCT02743741	Recruiting	Not Applicable	Lu-DOTATATE Treatment in Patients With 68Ga-DOTATATE Somatostatin Receptor PositiveNeuroendocrine Tumors	Lutetium-177 Octreotate	The proportion of patients who are progression-free using RECIST 1.1 criteria [Time frame: up to 12 months].
NCT02489604	Recruiting	2	Peptide Receptor Radionuclide Therapy (PRRT) With 177Lu-DOTATATE in Advanced Gastro-entero Pancreatic Neuroendocrine Tumors	177Lu-DOTATATE 25.9 GBq activity; 177Lu-DOTATATE 18.5 GBq activity	Disease control rate (DCR) [Time frame: up to 7 years].
NCT03478358	Recruiting	1	Treatment Using 177Lu-DOTA-EB-TATE in Patients with Advanced Neuroendocrine Tumors	177Lu-DOTA-EB-TATE 1; 177Lu-DOTA-TATE; 177Lu-DOTA-EB-TATE 2; 177Lu-DOTA-EB-TATE 3.	Change of standardized uptake value of 68Ga-DOTA-TATE before and after treatment in metastatic neuroendocrine tumors [Time frame: 1 year].
NCT00004074	Completed	1	Interleukin-12 and Trastuzumab in Treating Patients with Cancer That Has High Levels of HER2/Neu	Recombinant interleukin-12; ABI007/carboplatin/trastuzumab	Maximum tolerated dose (MTD) determined according to dose-limiting toxicities (DLTs), graded using the CTCAE v2.0 criteria.
NCT00005842	Completed	1	Trastuzumab Plus R115777 in Treating Patients with Advanced or Metastatic Cancer	Trastuzumab; tipifarnib	Determine the maximum tolerated dose of R115777 when administered with trastuzumab (Herceptin).

^1^ Last update from clinicaltrials.gov was on 26 August 2019.
